# Characterization of Capillary Zone Electrophoresis in Horses and Donkeys

**DOI:** 10.3390/vetsci13060513

**Published:** 2026-05-26

**Authors:** Carmen Davias, Francisco J. Mendoza, Adelaida De Las Heras, Alejandro Perez-Ecija

**Affiliations:** Department of Animal Medicine and Surgery, University of Cordoba, 14014 Cordoba, Spain; carmendaviasm@gmail.com (C.D.); v02hesaa@uco.es (A.D.L.H.); alejandro.perez.ecija@uco.es (A.P.-E.)

**Keywords:** donkeys, electrophoresis, horses, proteinogram

## Abstract

There are no capillary zone electrophoresis (CZE) comparative studies between horses and donkeys. Our objectives were to compare CZE results between both species, establish reference intervals (Ris), study age- and sex-related changes, determine the prevalence of bisalbuminemia, and correlate spectrophotometric albumin results with those calculated based on two different CZE methods. We performed CZE in 50 healthy horses and 49 healthy donkeys. There were significant differences (*p* < 0.03) in every protein fraction percentage and concentration (except for the β-globulins percentage) between both species, underlining the necessity of species-specific RIs. Considering the correlation to spectrophotometric results, the cathodal shoulder of the albumin should be kept in this fraction when performing CZE. Age- and sex-related differences in globulin fractions were noted in horses, but not in donkeys. Bisalbuminemia was a common finding in horses (36% of the animals), but was seldom observed in donkeys (4%). Further studies are warranted to investigate the clinical implications of these findings.

## Simple Summary

Serum protein electrophoresis is used in veterinary medicine to detect dysproteinemias and classify hyperglobulinemic cases. Capillary zone electrophoresis is the standard technique in human medicine and is nowadays used in large veterinary facilities. However, a detailed comparison between the results of this technique in horses and donkeys has not been reported. In this study, we established reference intervals for this technique in a large population of healthy horses and donkeys, demonstrating that there are significant differences between both species, as well as sex- and age-related differences in horses. Bisalbuminemia was found to be common in horses, but rare in donkeys.

## 1. Introduction

Multiple differences in clinical pathology parameters have been described between horses and donkeys, including discrepancies in hematology, biochemistry, and hemostatic reference ranges [1,2,3,4]. Thus, data extrapolation among equids can lead to misdiagnosis, unnecessary treatments, and increased expenses.

Serum protein electrophoresis (SPE) is the most commonly used technique for specific quantitation of serum protein fractions in veterinary medicine [5]. SPE allows the detection of qualitative abnormalities in major proteins in order to differentiate between causes of dysproteinemias, mainly those causing hyperglobulinemia such as monoclonal and polyclonal gammopathies [6,7]. In addition, although spectrophotometric methods are considered the gold standard, SPE can also be used to determine serum albumin concentrations based on serum total protein concentrations and the percentage of albumin [7]. To the authors’ knowledge, neither the correlation between these two methods, nor the effect of different albumin fractionations in the electrophoretogram (e.g., exclusion of the cathodal shoulder of the albumin peak) has been described in equids.

Several SPE techniques, such as agarose gel (AGE) and acetate cellulose (ACE) electrophoresis, are routinely performed in many veterinary facilities. However, capillary zone electrophoresis (CZE) is currently the standard technique in human medicine and is replacing these previous methods in many large veterinary laboratories [8]. CZE allows for a fast and efficient separation of proteins using small sample volumes [9]. Moreover, this method has superior discriminatory and resolution capabilities compared to either AGE or ACE and allows for the recognition of subtle abnormalities in protein migration, such as bisalbuminemia [10,11].

While reference intervals (RIs) for AGE and ACE are available in horses [5,12], to the authors’ knowledge, RIs for CZE have not been established in this species. Concerning donkeys, although several studies have reported AGE, ACE and CZE findings [6,13,14,15], a standardization of the limits for each protein fraction is not available, with some studies even lacking a graphic example of the electrophoretogram. Moreover, the adequacy of some recommendations concerning the boundaries between fractions in horses, such as the exclusion of the cathodal (towards the right) shoulder of the albumin peak in order to avoid falsely low α_1_-globulins when using AGE [7,16], is unknown.

Bisalbuminemia (or alloalbuminemia) is a rare condition characterized by the presence of two peaks in the albumin fraction in the electrophoretogram. In human medicine, this finding has been linked to point mutations, pathological conditions such as diabetes mellitus or rheumatoid arthritis, or drug administration [11,17,18]. While anecdotical reports of bisalbuminemia can be found in horses [19], the prevalence of this finding in equids or its relationship to any disease is still unknown.

Therefore, the objectives of this study were: (a) to establish the RIs for CZE in healthy horses and donkeys and study the effect of age and sex on these parameters; (b) to determine the correlation between serum albumin concentrations measured by spectrophotometry and those calculated by CZE in both species; and (c) to study the prevalence of bisalbuminemia in healthy horses and donkeys.

## 2. Materials and Methods

### 2.1. Animals and Determinations

Blood samples were collected into EDTA and serum separator tubes via jugular venipuncture from 49 donkeys (7.9 ± 0.6, range: 8 months–22 years old; 20 jacks—8 intact jacks and 12 geldings—and 29 jennies) and 50 horses (8.7 ± 0.6, range: 2–19 years old; 30 males—15 stallions and 15 geldings—and 20 mares) from farms located in different areas in Spain. Every sample was taken during the autumn. No animal younger than 6 months was included in this study. Forty-eight donkeys were of the Andalusian breed, and one was of the Catalonian breed. Regarding horse breeds, 21 were Andalusian, 25 were crossbreeds with Andalusian, 2 were Spanish Sport Horses, and 2 were Hannoverian. Samples with hemolysis or lipemia were excluded.

All animals were considered healthy based on clinical history, physical examination (heart and respiratory rates, temperature, mucous membrane color, capillary refill time, intestinal motility, and digital pulses) and a complete blood work profile. All animals received humane care in compliance with the Spanish Guide for the Care and Use of Laboratory Animals.

A complete CBC was performed in each animal using a Sysmex XN-1000 V analyzer (Sysmex Corporation, Kobe, Japan). After clotting, serum tubes were centrifuged at 3500 rpm for 5 min at 4 °C, and serum was aliquoted into 1.5 mL tubes and frozen at −20 °C until measurements. The total protein (TP) concentration was determined by the biuret method, and the albumin concentration was determined by the bromocresol green method (BCG), both using an automated analyzer (Spin 640 Plus, Spinreact, Barcelona, Spain). In order to confirm the absence of any disease, the following additional biochemical parameters were analyzed using spectrophotometry (Spin 640 Plus, Spinreact, Barcelona, Spain): glucose, triglycerides, cholesterol, urea, creatinine, total bilirubin, alkaline phosphatase, gamma-glutamyl transferase, glutamate dehydrogenase, aspartate aminotransferase, creatine kinase, and lactate dehydrogenase. Globulin concentrations and the albumin:globulin ratio (AGR) were calculated according to previously reported formulas [20].

CZE was performed using the Capillarys 2 analyzer (SEBIA, Issy les Moulineux, France) in veterinary mode (software version: Phoresis 9.1.5). The protein fractions and the electrophoretic curves were visually inspected and adjusted as previously described [7]. Briefly, the boundary between albumin and α_1_-globulins was separated following two different approaches: one maintaining the cathodal (towards the right) boundary of the albumin peak in this fraction (CZE1), and the other one including this region inside the α_1_-globulins (CZE2). Alpha-globulins were separated into α_1_ and α_2_ globulins (this last fraction appeared anodal to the mid-point of the tracing). The beta-globulin fraction was recognized as the fraction immediately cathodal to the mid-point of the tracing, while the diffuse staining at the end of the electrophoretogram was determined to correspond to gamma-globulins. All samples were analyzed by the same person (A.P-E). The concentration of each protein fraction was calculated based on the percentages obtained and serum TP concentrations.

The presence of bisalbuminemia was recorded, and each case was classified as biphasic (with two distinctive curves sharing their bases) or bifid (with two distinctive peaks sharing at least two-thirds of their base) by two different observers (A.P-E and F.J.M).

### 2.2. Statistical Analysis

The results are expressed as the mean ± standard error (SE) or median and interquartile range (IQR: 75th–25th percentiles) according to normality. Normality was assessed by the Shapiro–Wilk test. Percentiles were calculated using Tukey’s Hinges test. Outlier values (lower or upper quartile ± 1.5 times the interquartile range) were determined by Huber’s test. Since the mean 5% trimmed did not significantly change the final results, the outlier values were not excluded from statistical analysis. Differences between two groups (horses versus donkeys; effect of sex) were studied using an unpaired *t*-test or a Mann–Whitney test.

Donkeys and horses were grouped into the following age groups: Group 1: <5 years old; Group 2: 5–10 years old; and Group 3: >10 years old. Age groups were compared using either the Kruskal–Wallis test with Dunn’s post hoc test or a one-way ANOVA with Tukey’s post hoc test, depending on the normality.

Agreement between methods for albumin determination (spectrophotometry vs. CZE1 or CZE2) was evaluated using Passing–Bablok regression analysis and Bland–Altman difference plots. Agreement was considered good when two conditions were fulfilled: the 95% confidence interval (CI) included the value 0 for the intercept in Bland–Altman, and the value 1 for the slope in the Passing–Bablok regression.

Inter-observer agreement for the presence and classification of bisalbuminemia was calculated using Cohen’s kappa test. Reference intervals were obtained following the recommendations of the American Society of Veterinary Clinical Pathology (ASVCP), with either a robust or a non-parametric method, using a dedicated software (Reference Value Advisor v. 2.1. freeware. This is available at http://www.biostat.envt.fr/reference-value-advisor/ (accessed on 23 January 2026) with findings presented as two-sided 90% CIs [21].

Statistical analyses were performed using specific statistical packages (IBM SPSS Statistics 27, IBM Corporation, Armonk, NY, USA; MedCalc 23.2.0, Ostend, Belgium; GraphPad Prism 9, San Diego, CA, USA). Values with *p* < 0.05 were considered significant.

## 3. Results

### 3.1. Agreement Among Methods for Serum Albumin Concentration Measurement

**Figure 1:**
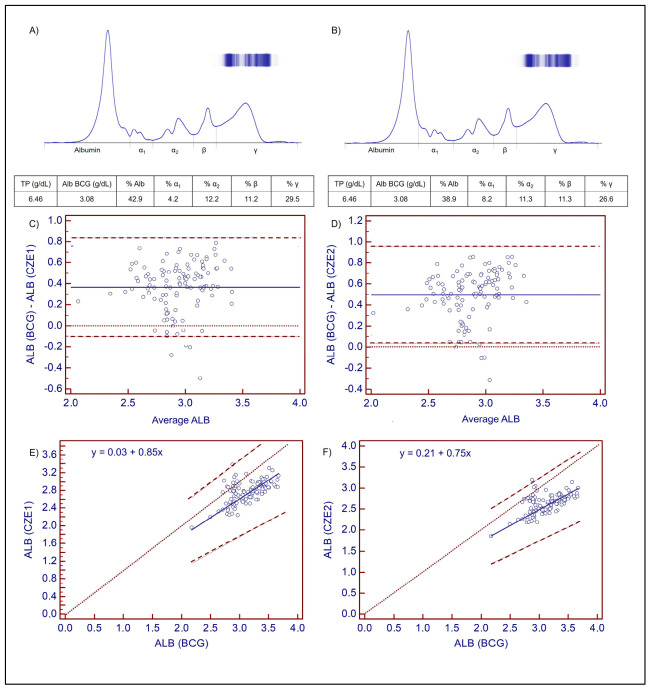
Comparison between methods for albumin determination in equids. Electrophoretogram and fraction results of the same horse, considering the cathodal shoulder of the albumin as part of this fraction CZE1 (**A**) and including this shoulder as part of α1-globulins CZE2 (**B**). Bland–Altman plots comparing spectrophotometric (BCG) albumin results vs. CZE1 (**C**) and CZE2 (**D**). Passing–Bablok regression comparing BCG albumin with CZE1 (**E**) and CZE2 (**F**). In the Bland-Altman plots, X-axes represent the averages for the two measurements of both methods, and the Y-axes indicate the difference of both assessments. The solid blue line represents the mean difference (bias), with the solid brown line being equal to zero, and the dashed lines indicating the limits of agreement. In the Passing-Bablok regression, the solid blue line represents the regression line, the dashed lines represent the 95% confidence intervals, and the solid brown line represents the identity line. The regression equation is detailed into the graph.

Findings on the agreement between albumin concentrations measured by spectrophotometry and CZE1 or CZE2 are compiled in Figure 1.

Agreement was good for CZE1 (albumin fraction complete, including its cathodal region), with the Bland–Altman plot showing an intercept of 0.03 (95% CI between −0.43 and 0.43) and Passing–Bablok regression showing a slope of 0.85 (95% CI between 0.73 and 1.02). In contrast, this agreement was poor when compared with CZE2, where requirements regarding the CIs were not fulfilled in the Passing–Bablok regression (slope 0.75, 95% CI between 0.65 and 0.89). In both instances, there was a negative bias in the albumin concentration calculated using CZE compared to spectrophotometry, although this difference was more marked with CZE2 (Figure 1).

### 3.2. CZE Data and RIs in Healthy Donkeys and Horses

**Figure 2:**
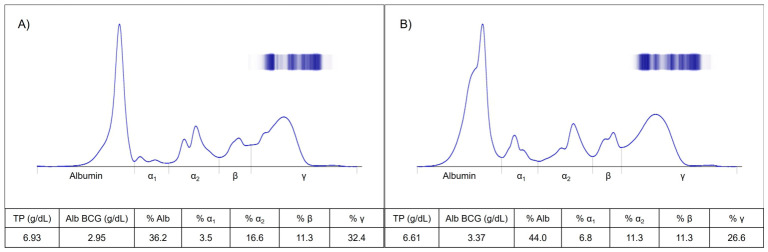
Examples of capillary zone electrophoresis curves in one healthy donkey (**A**) and one healthy horse (**B**), along with their protein concentrations and protein fraction percentages. Note that the “kinked” appearance of albumin in this horse was not considered a case of bisalbuminemia. α_1_, α_1_-globulins; α_2_, α_2_-globulins; β, β-globulins; γ, γ-globulins; Alb, albumin; BCG, bromocresol green; TP, total proteins.

According to the results obtained in the previous objective, the limits of the albumin fraction were drawn using the CZE1 approach (Figure 2). Two distinct fractions were recognized in every animal in the α-globulins area (α_1_- and α_2_-globulins). The boundary between β- and γ-globulins was also easily identified in most animals (Figure 2). No other subdivisions (e.g., β_1_- or β_2_-globulins) were recognized in the electrophoretograms.

The percentages of every protein fraction, the concentrations of total proteins, albumin, and globulins measured by spectrophotometry, as well as the calculated AGR, are compiled in Table 1. Healthy donkeys had significantly (*p* < 0.05) lower albumin and α_1_-globulins, as well as higher α_2_ and γ-globulins (both in percentage and concentration) than healthy horses. No differences were observed for β-globulin percentages between both species, although concentrations were significantly higher (*p* < 0.05) in donkeys (Table 1). Regarding spectrophotometry results, albumin concentration was lower and total proteins and globulin concentrations were higher (*p* < 0.05) in donkeys compared to horses (Table 1). AGR was lower (*p* < 0.05) in donkeys than in horses (Table 1).

The RIs for each protein fraction (and their concentrations) in healthy donkeys and horses using CZE are presented in Table 2.

### 3.3. Effect of Age on CZE Results in Healthy Donkeys and Horses

Regarding spectrophotometry data, no statistically significant differences were found among age groups for serum total proteins, albumin, globulins, and the AGR for either donkeys or horses. For CZE, no differences were found between donkey groups (Table 3). Younger horses (<5 years old) showed significantly higher (*p* < 0.05) concentrations of α_2_-globulins than the rest of the groups and significantly higher (*p* < 0.05) concentrations and percentages of β-globulins than horses between 5 and 10 years old (Table 3).

### 3.4. Effect of Sex on CZE Results in Healthy Donkeys and Horses

When results obtained by spectrophotometry were compared between sex groups, no significant differences were found in serum total proteins, albumin, globulins, and AGR in either donkeys or horses (Table 4). Regarding CZE results, no differences were found between jennies and jacks. Mares showed significantly (*p* < 0.05) higher α_2_-globulin concentrations and β-globulin percentages and concentrations than males (Table 4).

### 3.5. Prevalence of Bisalbuminemia in Serum Samples from Healthy Horses and Donkeys

**Figure 3:**
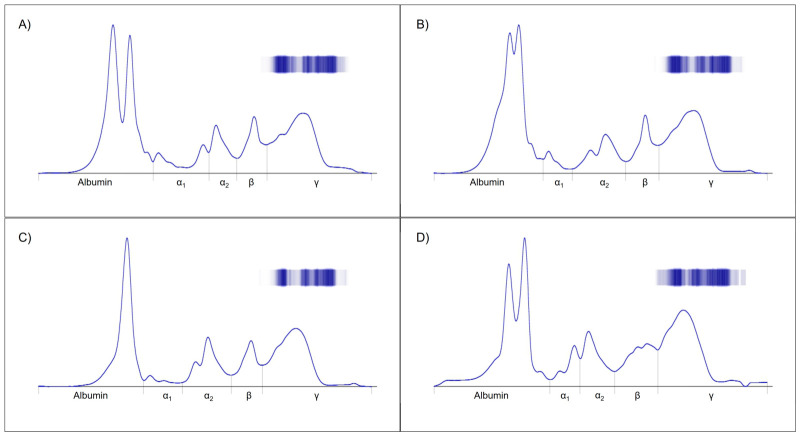
Examples of bisalbuminemia in CZE in equids. (**A**) Biphasic curve in a healthy horse, with two distinctive albumin peaks sharing only their bases. (**B**) A Bifid curve in a healthy horse, where both peaks share at least two-thirds of their base. Non-bisalbuminemic healthy donkey (**C**) versus another healthy donkey showing a biphasic bisalbuminemic pattern (**D**). α_1_, α_1_-globulins; α_2_, α_2_-globulins; β, β-globulins; γ, γ-globulins.

Bisalbuminemia (Figure 3) was found in 18 out of 50 healthy horses (with a prevalence of 36%). Fourteen cases were considered biphasic, and four cases were classified as bifid. The distribution of bisalbuminemia was fairly even between age and sex groups, although this change was more common in males (12/30, 40%) compared to females (6/20, 30%) and appeared to increase with age (2/11 or 18.2% in Group 1; 7/19 or 36.8% in Group 2; and 9/20 or 45% of the animals in Group 3).

On the other hand, this condition was only found in 2/49 healthy donkeys (4.08% of the samples; one 3-year-old jenny and one 2-year-old jack), with both cases classified as biphasic (Figure 3). Cohen’s kappa test result was high (κ  =  0.94 ± 0.02) between both observers.

## 4. Discussion

The presence of significant differences in almost every protein fraction (both in percentages and concentrations) between species denotes the existence of underlying physiological variations between these parameters and justifies the need for species-specific RIs in equids. In our study, donkeys showed significantly lower albumin and α_1_-globulin levels, but higher α_2_- and γ-globulin percentages and concentrations than horses.

Serum protein electrophoresis is not sufficiently standardized in equids, with only one report comparing results between horses and donkeys using AGE [6]. This is the first study establishing RIs for CZE in healthy horses and donkeys following the recommendations of the ASVCP. Using this technique, horses had lower albumin but higher α_1_- and γ-globulin percentages, with similar α_2_- and β-globulin percentages compared to a previous report using AGE [5]. In contrast, both albumin and γ-globulin percentages were higher compared to other older AGE reports [22,23]. These inter-study variations could be due to technical dissimilarities (CZE versus AGE) and/or differences in protein fractionation, with some authors dividing β- (β_1_- and β_2_-globulins) and/or γ- (γ_1_- and γ_2_) globulins and even differentiating between up to five fractions in α-globulins. We could not clearly differentiate those subdivisions in our curves and, as previously recommended, we did not separate fractions lacking correspondence to a visible gap in the electrophoretogram [5]. Furthermore, fast migrating α_1_-globulins in equine serum can cause a cathodal shoulder in the albumin, which has led some authors to recommend excluding this area for albumin determination [7]. However, we found that this method under-represented albumin concentrations when compared with spectrophotometric results, even causing a poor agreement between techniques. When this shoulder was included in the albumin fraction, the agreement between techniques was good (although a negative bias was apparent in CZE). Similar conclusions were reached by previous authors using AGE [5].

Our percentages are similar to those in the only study reporting CZE in donkeys [15]. While these authors described the presence of three small peaks in α_1_-globulins as a specific feature in donkeys, our results did not consistently agree with this finding (1/49 of our donkeys showed three distinct peaks; 12/49 showed two peaks; and 36/49 showed one peak). Moreover, horses also presented variability in the number of peaks in α_1_-globulins (2/50 horses with three peaks; 15/50 horses with three peaks; and 33/50 horses with one peak). Whether this discrepancy between studies is due to technical variations between analyzers and/or breed-related differences is unknown. Compared to a previous study using AGE in donkeys [6], we observed higher γ-globulin but lower β-globulin percentages. On the other hand, previous studies in donkeys using ACE reported similar albumin and γ-globulin levels, but different α- and β-globulin percentages compared to our results [13,14]. These variations could be related to different fractionation of proteins, the effect of different media, and/or genetic factors.

Concerning spectrophotometric results for serum total proteins, albumin, globulins and the AGR, our results are within the reference ranges reported for both species [4].

The effect of age on SPE parameters in equids is controversial, with discrepancies among studies, probably due to differences between techniques, variations in age grouping and various approaches to protein fraction identification. Previous studies have described higher α_2_-globulin concentrations in younger donkeys compared to older ones [13,15]. We also observed this trend, but the difference was not statistically significant. Other age-related variations in fractions such as albumin and β- or γ-globulins, described by some authors in donkeys, were not apparent in our results [13,15]. Regarding horses, one previous study using ACE reported lower albumin and higher α_1_- and α_2_-globulin concentrations in younger animals compared to older ones [24]. Our results partially agree with this report, showing a similar effect in α_2_-globulins, but no variation in albumin or α_1_-globulins was detected. Moreover, we also observed higher β-globulin percentages and concentrations in younger horses compared to animals between 5 and 10 years old, which was not described in that study. One limitation of our results concerning age is the inclusion of every animal older than 10 years in a single group. This decision was made in order to homogenize the size of the groups. Moreover, we did not observe any significant age-related variation in any parameter within this group that could justify further subdivisions. Previous SPE reports studying the effect of age in donkeys also used large age ranges in the adult or older group, such as 10–17 or even 3–18 years old [13,15]. Only horses between 15 and 20 years old were included in the work reporting age-related differences in this species, without any animals between 11 and 15 years old [24].

Few reports have focused on the effect of sex on SPE parameters in equids. Similar to our findings, no sex-related differences were found in donkeys in a previous report using AGE [6]. On the other hand, we found significant differences between mares (higher α_2_-globulin concentrations and β-globulin concentrations and percentages) and males, contrary to a previous AGE study [6]. This difference was limited to CZE parameters, since spectrophotometric results were similar between sexes. Whether this finding is related to hormonal influences or the effect of unbalanced representations of ages in our sex group should be further studied.

Bisalbuminemia is a rare benign condition in human beings, which can be either hereditary (a point mutation of the albumin gene inherited in an autosomal codominant pattern) or acquired [10,17]. Several pathologies, such as diabetes mellitus, rheumatoid arthritis, multiple myeloma, and drugs, have been associated with this incidental finding [25,26]. Due to its inherently higher resolution, CZE is the best method to detect bisalbuminemia, with several reports showing the inability of AGE or ACE to recognize this subtle change [27]. Similarly, it has been reported that CZE was able to detect hereditary bisalbuminemia in two families of bottlenose dolphins, whereas AGE failed [17]. A spurious peak, similar to bisalbuminemia, has been described in hypercholesterolemic dogs [28]. Although bisalbuminemia has been described in horses as a common but not invariable finding, both in healthy and sick animals [19], no data about its prevalence is available. Moreover, no study has reported its presence in donkeys.

According to our results, bisalbuminemia is a common finding in horses, with more than a third of healthy animals (both sexes and every age group) showing this change. We did not find any apparent difference between cases classified as bifid or biphasic, and this finding was not correlated to any change in CZE protein percentages or spectrophotometric results. On the other hand, bisalbuminemia was rare in donkeys, with only two healthy animals showing it. While this variation could be a species-specific idiosyncrasy in equids, it would be necessary to perform additional genetic analysis to confirm this hypothesis. Further studies are warranted to determine the clinical relevance of this finding.

The present study has some limitations. For example, the number of animals in each sex and age group was not identical, which could have affected our RIs and/or findings. Additionally, since some of our age and sex cohorts were small, differences between these groups should be taken with caution. Our donkeys and horses were based in Spain and mostly of Spanish breeds. While differences in serum protein fractions have been reported among different breeds in dogs [29], no significant variations were found between thoroughbreds and draught horses in a previous study [5]. Nonetheless, we cannot discard the presence of breed-, environmental- or geographical-related idiosyncrasies in our populations. Thus, caution should be taken when extrapolating our RIs to other equid breed populations. Since hemolysis and lipemia have been reported to interfere with protein fraction concentrations in CZE in dogs [30], we excluded any sample with these findings. Further studies on the possible effects of these interferants in equids are necessary.

## 5. Conclusions

This study is the first to describe RIs for CZE in donkeys and horses. Since CZE results were significantly different between healthy horses and donkeys, species-specific RIs should be used. Mares showed higher α2- and β-globulin concentrations than male horses, and a similar variation was observed between younger horses (<5 years old) and those between 5 and 10 years old. Bisalbuminemia appears to be a common finding in healthy horses but is only rarely seen in donkeys.

## Figures and Tables

**Table 1:** Protein fractions and concentrations in healthy donkeys and horses analyzed by spectrophotometry and capillary zone electrophoresis.

	Parameter	Donkeys (*n* = 49)	Horses (*n* = 50)	*p* Values
Spectro	Total proteins (g/dL)	6.87 (0.26) * (6.82–6.97)	6.57 (0.59) (6.36–6.68)	<0.001
	Albumin (g/dL)	2.94 (0.30) * (2.89–3.02)	3.33 (0.32) (3.21–3.38)	<0.001
	Globulins (g/dL)	3.96 (0.36) * (3.89–4.04)	3.31 (0.53) (3.12–3.45)	<0.001
	AGR	0.73 (0.14) * (0.72–0.75)	0.98 (0.27) (0.92–1.08)	<0.001
CZE	Albumin (%)	38.7 ± 0.59 * (37.4–39.8)	43.3± 0.57 (42.2–44.6)	<0.001
	Albumin (g/dL)	2.65 ± 0.04 * (2.57–2.73)	2.83 ± 0.03 (2.78–2.89)	<0.001
	α_1_-globulins (%)	4.57 ± 0.11 * (4.34–4.80)	6.57 ± 0.27 (6.03–7.11)	<0.001
	α_1_-globulins (g/dL)	0.31 ± 0.01 * (0.29–0.33)	0.43 ± 0.02 (0.39–0.46)	<0.001
	α_2_-globulins (%)	15.0 ± 0.33 * (14.4–15.7)	11.5 ± 0.28 (10.9–12.1)	<0.001
	α_2_-globulins (g/dL)	1.03 ± 0.02 * (0.99–1.08)	0.76 ± 0.02 (0.71–0.80)	<0.001
	β-globulins (%)	12.9 ± 0.39 (12.1–13.7)	12.0 ± 0.41 (11.2–12.8)	0.119
	β-globulins (g/dL)	0.89 ± 0.03 * (0.83–0.95)	0.79 ± 0.03 (0.72–0.85)	0.03
	γ-globulins (%)	29.9 (5.55) * (28.1–31.3)	26.4 (4.25) (25.5–28.0)	<0.001
	γ-globulins (g/dL)	2.02 (0.33) * (1.90–2.09)	1.76 (0.43) (1.64–1.87)	<0.001

Data are expressed as mean ± SE or median (IQR, interquartile range) and 95% confidence intervals of mean or median, depending on distribution. AGR: albumin:globulin ratio; CZE: capillary zone electrophoresis; Spectro: spectrophotometry. * *p* < 0.05 vs. horses.

**Table 2:** Reference intervals for CZE protein fractions and concentrations with 90% CIs of upper and lower RIs in healthy donkeys and horses.

	Parameter	Lower Reference Limit (CI 90%)	Upper Reference Limit (CI 90%)
Donkeys (*n* = 49)	Albumin (%)	31.6 (31.5–32.8)	49.1 (45.4–49.2)
	Albumin (g/dL)	2.0 (2.0–2.2)	3.3 (3.1–3.4)
	α_1_-globulins (%)	3.2 (3.1–3.4)	6.2 (5.9–6.3)
	α_1_-globulins (g/dL)	0.2 (0.2–0.2)	0.4 (0.4–0.4)
	α_2_-globulins (%)	10.3 (10.1–11.4)	20.2 (19.4–20.2)
	α_2_-globulins (g/dL)	0.7 (0.7–0.8)	1.4 (1.3–1.4)
	β-globulins (%)	7.9 (7.7–8.8)	19.3 (17.0–19.7)
	β-globulins (g/dL)	0.5 (0.5–0.6)	1.4 (1.3–1.4)
	γ-globulins (%)	16.1 (15.5–19.6)	36.9 (33.3–37.8)
	γ-globulins (g/dL)	1.0 (0.9–1.3)	2.7 (2.6–2.7)
Horses (*n* = 50)	Albumin (%)	36.2 (36.1–37.9)	54.2 (50.2–55.0)
	Albumin (g/dL)	2.4 (2.4–2.5)	3.3 (3.1–3.3)
	α_1_-globulins (%)	3.5 (3.4–3.8)	11.3 (9.3–12.0)
	α_1_-globulins (g/dL)	0.2 (0.2–0.3)	0.7 (0.6–0.8)
	α_2_-globulins (%)	7.0 (7.0–8.2)	15.1 (14.4–15.3)
	α_2_-globulins (g/dL)	0.4 (0.4–0.5)	1.1 (1.0–1.1)
	β-globulins (%)	7.6 (7.4–8.5)	21.9 (17.9–23.0)
	β-globulins (g/dL)	0.5 (0.5–0.6)	1.6 (1.2–1.7)
	γ-globulins (%)	16.0 (15.2–21.8)	34.0 (32.0–34.2)
	γ-globulins (g/dL)	1.0 (0.9–1.2)	2.2 (2.1–2.2)

CI, confidence interval.

**Table 3:** CZE and spectrophotometry results in healthy donkeys and horses grouped by age.

	Parameter	Group 1: <5 Years Old	Group 2: 5–10 Years Old	Group 3: >10 Years Old
		Donkeys (*n* = 49)		
		(*n* = 15)	(*n* = 19)	(*n* = 15)
Spectro	Total proteins (g/dL)	6.85 (0.39) (6.60–7.01)	6.90 (0.25) (6.74–6.99)	6.87 (0.20) (6.82–7.02)
	Albumin (g/dL)	2.99 ± 0.05 (2.87–3.11)	2.89 ± 0.06 (2.75–3.02)	3.01 ± 0.03 (2.93–3.08)
	Globulins (g/dL)	3.91 ± 0.11 (3.67–4.16)	4.01 ± 0.06 (3.88–4.14)	3.95 ± 0.09 (3.77–4.14)
	AGR	0.72 (0.12) (0.69–0.90)	0.73 (0.16) (0.69–0.74)	0.73 (0.11) (0.72–0.83)
CZE	Albumin (%)	37.8 ± 0.94 (35.8–39.8)	38.1 ± 0.83 (36.4–39.8)	38.8 ± 0.92 (36.8–40.7)
	Albumin (g/dL)	2.61 ± 0.06 (2.49–2.73)	2.63 ± 0.07 (2.48–2.78)	2.69 ± 0.05 (2.59–2.80)
	α_1_-globulins (%)	4.85 ± 0.20 (4.41–5.29)	4.40 ± 0.18 (4.03–4.77)	4.50 ± 0.18 (4.12–4.88)
	α_1_-globulins (g/dL)	0.33 ± 0.02 (0.30–0.37)	0.30 ± 0.01 (0.28–0.32)	0.31 ± 0.01 (0.29–0.33)
	α_2_-globulins (%)	15.6 ± 0.70 (14.4–17.3)	14.3 ± 0.41 (13.5–15.2)	14.3 ± 0.41 (13.5–15.2)
	α_2_-globulins (g/dL)	1.10 ± 0.05 (0.99–1.20)	0.99 ± 0.03 (0.93–1.05)	1.00 ± 0.03 (0.93–1.07)
	β-globulins (%)	13.2 ± 0.71 (11.7–14.7)	13.2 ± 0.67 (11.8–14.5)	11.4 ± 0.63 (10.1–12.8)
	β-globulins (g/dL)	0.92 ± 0.06 (0.79–1.04)	0.91 ± 0.05 (0.81–1.01)	0.80 ± 0.05 (0.69–0.90)
	γ-globulins (%)	28.3 ± 1.23 (25.6–30.8)	29.9 ± 0.57 (28.8–31.1)	30.9 ± 0.68 (29.5–32.4)
	γ-globulins (g/dL)	1.95 ± 0.10 (1.75–2.15)	2.07 ± 0.05 (1.97–2.16)	2.16 ± 0.06 (2.03–2.28)
		Horses (*n* = 50)		
		(*n* = 11)	(*n* = 19)	(*n* = 20)
Spectro	Total proteins (g/dL)	6.78 ± 0.15 (6.45–7.11)	6.47 ± 0.06 (6.35–6.60)	6.52 ± 0.12 (6.28–6.76)
	Albumin (g/dL)	3.28 ± 0.09 (3.09–3.47)	3.28 ± 0.06 (3.17–3.39)	3.31 ± 0.05 (3.21–3.41)
	Globulins (g/dL)	3.50 ± 0.12 (3.22–3.77)	3.20 ± 0.07 (3.05–3.34)	3.21 ± 0.13 (2.93–3.48)
	AGR	0.95 ± 0.05 (0.85–1.05)	1.03 ± 0.03 (0.97–1.11)	1.07 ± 0.05 (0.96–1.18)
CZE	Albumin (%)	40.9 ± 0.56 (39.7–42.1)	42.7 ± 0.80 (41.0–44.4)	44.4 ± 1.10 (42.1–46.7)
	Albumin (g/dL)	2.91 ± 0.05 (2.82–2.99)	2.76 ± 0.05 (2.66–2.86)	2.89 ± 0.04 (2.81–2.98)
	α_1_-globulins (%)	6.70 ± 0.51 (5.56–7.84)	7.32 ± 0.35 (6.60–8.06)	6.07 ± 0.49 (5.04–7.10)
	α_1_-globulins (g/dL)	0.48 ± 0.04 (0.39–0.56)	0.47 ± 0.02 (0.43–0.52)	0.39 ± 0.03 (0.33–0.46)
	α_2_-globulins (%)	12.9 ± 0.49 (11.9–14.1)	11.6 ± 0.56 (10.4–12.7)	11.5 ± 0.52 (10.4–12.6)
	α_2_-globulins (g/dL)	0.93 ± 0.04 (0.83–1.02)	0.75 ± 0.03 * (0.67–0.82)	0.76 ± 0.04 * (0.67–0.85)
	β-globulins (%)	14.5 ± 1.21 (11.8–17.2)	11.5 ± 0.59 * (10.3–12.7)	12.6 ± 0.75 (11.0–14.1)
	β-globulins (g/dL)	1.04 ± 0.09 (0.82–1.24)	0.75 ± 0.04 * (0.66–0.82)	0.84 ± 0.06 (0.71–0.96)
	γ-globulins (%)	24.9 ± 1.03 (22.6–27.1)	26.9 ± 0.93 (24.9–28.8)	25.4 ± 0.83 (23.6–27.1)
	γ-globulins (g/dL)	1.77 ± 0.08 (1.60–1.93)	1.75 ± 0.07 (1.60–1.88)	1.68 ± 0.08 (1.52–1.84)

Data are expressed as mean ± SE or median (IQR, interquartile range) and 95% confidence intervals of mean or median, according to the distribution. AGR: albumin:globulin ratio; CZE: capillary zone electrophoresis; Spectro: spectrophotometry. * *p* < 0.05 vs. Group 1.

**Table 4:** CZE and spectrophotometry results in healthy donkeys and horses grouped by sex.

	Parameter	Males	Females
		Donkeys (*n* = 49)	
		(*n* = 20)	(*n* = 29)
Spectro	Total proteins (g/dL)	6.87 (0.29) (6.71–7.00)	6.84 (0.39) (6.70–7.01)
	Albumin BCG (g/dL)	2.91 (0.28) (2.79–3.07)	2.95 (0.35) (2.84–3.15)
	Globulins (g/dL)	4.01 (0.33) (3.88–4.21)	3.95 (0.42) (3.73–4.09)
	AGR	0.73 (0.10) (0.64–0.75)	0.73 (0.18) (0.71–0.85)
CZE	Albumin (%)	37.2 ± 0.89 (35.3–39.0)	37.9 ± 0.68 (36.4–39.5)
	Albumin (g/dL)	2.57 ± 0.07 (2.43–2.72)	2.57 ± 0.05 (2.48–2.67)
	α_1_-globulins (%)	4.46 ± 0.16 (4.13–4.80)	4.55 ± 0.14 (4.22–4.88)
	α_1_-globulins (g/dL)	0.31 ± 0.01 (0.28–0.33)	0.31 ± 0.01 (0.29–0.33)
	α_2_-globulins (%)	14.5 ± 0.59 (13.3–15.8)	15.8 ± 0.34 (15.0–16.6)
	α_2_-globulins (g/dL)	1.00 ± 0.04 (0.92–1.09)	1.07 ± 0.03 (1.02–1.14)
	β-globulins (%)	13.1 ± 0.73 (11.6–14.6)	13.4 ± 0.43 (12.5–14.3)
	β-globulins (g/dL)	0.91 ± 0.06 (0.79–1.04)	0.91 ± 0.03 (0.84–0.99)
	γ-globulins (%)	30.7 ± 0.78 (29.1–32.4)	28.3 ± 0.33 (26.4–30.0)
	γ-globulins (g/dL)	2.13 ± 0.07 (1.99–2.27)	1.93 ± 0.07 (1.78–2.07)
		Horses (*n* = 58)	
		(*n* = 30)	(*n* = 20)
Spectro	Total proteins (g/dL)	6.46 ± 0.06 (6.33–6.58)	6.61 ± 0.11 (6.39–6.85)
	Albumin (g/dL)	3.24 ± 0.04 (3.17–3.32)	3.30 ± 0.06 (3.18–3.42)
	Globulins (g/dL)	3.21 ± 0.06 (3.07–3.35)	3.31 ± 0.13 (3.05–3.58)
	AGR	1.03 ± 0.03 (0.97–1.09)	1.03 ± 0.05 (0.92–1.14)
CZE	Albumin (%)	43.7 ± 0.76 (42.2–45.3)	42.5 ± 0.93 (40.6–44.4)
	Albumin (g/dL)	2.81 ± 0.04 (2.73–2.91)	2.86 ± 0.03 (2.80–2.94)
	α_1_-globulins (%)	6.86 ± 0.38 (6.09–7.64)	6.26 ± 0.36 (5.50–7.03)
	α_1_-globulins (g/dL)	0.44 ± 0.02 (0.39–0.49)	0.43 ± 0.03 (0.37–0.48)
	α_2_-globulins (%)	11.2 ± 0.41 (10.4–12.1)	12.3 ± 0.34 (11.6–13.0)
	α_2_-globulins (g/dL)	0.72 ± 0.03 (0.67–0.78)	0.84 ± 0.03 * (0.77–0.91)
	β-globulins (%)	11.3 ± 0.44 (10.5–12.3)	14.2 ± 0.88 * (12.4–16.1)
	β-globulins (g/dL)	0.73 ± 0.03 (0.67–0.80)	0.98 ± 0.07 * (0.83–1.13)
	γ-globulins (%)	26.8 ± 0.71 (25.4–28.3)	24.6 ± 0.77 (23.1–26.3)
	γ-globulins (g/dL)	1.73 ± 0.05 (1.63–1.85)	1.67 ± 0.06 (1.54–1.81)

Data are expressed as mean ± SE or median (IQR, interquartile range) and 95% confidence intervals of mean or median, according to the distribution. AGR: albumin:globulin ratio; CZE: capillary zone electrophoresis; Spectro: spectrophotometry. * *p* < 0.05 vs. males.

## Data Availability

The original contributions presented in this study are included in the article. Further inquiries can be directed to the corresponding author.

## References

[1] Perez-Ecija A., Gonzalez-De Cara C.A., Aguilera-Aguilera R., Estepa J.C., Rubio M.D., Mendoza F.J. (2014). Comparison of donkey hemogram using the LaserCyte hematology analyzer, an impedance system, and a manual method. Vet. Clin. Pathol..

[2] Perez-Ecija A., Mendoza F.J. (2017). Characterisation of clotting factors, anticoagulant protein activities and viscoelastic analysis in healthy donkeys. Equine Vet. J..

[3] Perez-Ecija A., Buzon-Cuevas A., Aguilera-Aguilera R., Gonzalez-De Cara C., Mendoza Garcia F.J. (2021). Reference intervals of acute phase proteins in healthy Andalusian donkeys and response to experimentally induced endotoxemia. J. Vet. Intern. Med..

[4] Goodrich E.L., Behling-Kelly E. (2019). Clinical Pathology of Donkeys and Mules. Vet. Clin. N. Am. Equine Pract..

[5] Riond B., Wenger-Riggenbach B., Hofmann-Lehmann R., Lutz H. (2009). Serum protein concentrations from clinically healthy horses determined by agarose gel electrophoresis. Vet. Clin. Pathol..

[6] Cavalcante P.H., Silva A.C., Sakamoto S., Soto-Blanco B. (2012). Serum protein fractions in Brazilian-breed donkeys using agarose gel electrophoresis. Turk. J. Vet. Anim. Sci..

[7] Friedrichs K.R., Scott M.A., Stockham S.L., Stockham S.L., Scott M.A. (2025). Proteins. Fundamentals of Veterinary Clinical Pathology.

[8] Giordano A., Paltrinieri S. (2010). Interpretation of capillary zone electrophoresis compared with cellulose acetate and agarose gel electrophoresis: Reference intervals and diagnostic efficiency in dogs and cats. Vet. Clin. Pathol..

[9] Clark R., Katzmann J.A., Wiegert E., Namyst-Goldberg C., Sanders L., Oda R.P., Kyle R.A., Landers J.P. (1996). Rapid capillary electrophoretic analysis of human serum proteins: Qualitative comparison with high-throughput agarose gel electrophoresis. J. Chromatogr. A..

[10] Avgoustou E., Kounatidis D., Vallianou N.G., Karampela I., Stratigou T., Dalamaga M. (2024). Incidental detection of hereditary bisalbuminemia in a patient with positive DAT coombs: A case-based review. Metabol. Open.

[11] Jaeggi-Groisman S.E., Byland C., Gerber H. (2000). Improved sensitivity of capillary electrophoresis for detection of bisalbuminemia. Clin. Chem..

[12] Kaneko J.J., Harvey J.W., Bruss M.L. (2008). Clinical Biochemistry of Domestic Animals.

[13] Dezzutto D., Barbero R., Valle E., Giribaldi M., Raspa F., Biasato I., Cavallarin L., Bergagna S., McLean A., Gennero M.S. (2018). Observations of the Hematological, Hematochemical, and Electrophoretic Parameters in Lactating Donkeys (*Equus asinus*). J. Equine Vet. Sci..

[14] Alberghina D., Fazio F., Arfuso F., Sciano S., Zumbo A., Piccione G. (2013). Reference Intervals of Serum Protein Concentrations from Clinically Healthy Female Ragusana Donkeys (Equus asinus) Determined by Cellulose Acetate Electrophoresis. J. Equine Vet. Sci..

[15] Caldin M., Furlanello T., Solano-Gallego L., De Lorenzi D., Carli E., Tasca S., Lubas G. (2005). Reference ranges for haematology, biochemical profile and electrophoresis in a single herd of Ragusana donkeys from Sicily (Italy). Comp. Clin. Pathol..

[16] Erickson E.D. Alpha globulins of equine serum. Proceedings of the First International Symposium on Equine Hematology (American Association of Equine).

[17] Gili C., Bonsembiante F., Bonanni R., Giordano A., Ledda S., Beffagna G., Paltrinieri S., Sommer M., Gelain M.E. (2016). Detection of hereditary bisalbuminemia in bottlenose dolphins (Tursiops truncatus, Montagu 1821): Comparison between capillary zone and agarose gel electrophoresis. BMC Vet. Res..

[18] Chhabra S., Bansal F., Saikia B., Minz R.W. (2013). Bisalbuminemia: A rarely encountered protein anomaly. J. Lab. Physicians.

[19] eClinPath.com, Cornell University. https://eclinpath.com/ngg_tag/bisalbumin/.

[20] Pires M.J., Cotovio M., Queiroga F., Pires C.A., Silvestre-Ferreira A.C. (2025). Reference Intervals for Biochemical Analytes in Clinically Healthy Adult Lusitano Horses. Vet. Sci..

[21] Friedrichs K.R., Harr K.E., Freeman K.P., Szladovits B., Walton R.M., Barnhart K.F., Blanco-Chavez J., American Society for Veterinary Clinical Pathology (2012). ASVCP reference interval guidelines: Determination of de novo reference intervals in veterinary species and other related topics. Vet. Clin. Pathol..

[22] Kristensen F., Firth E.C. (1977). Analysis of serum proteins and cerebrospinal fluid in clinically normal horses, using agarose electrophoresis. Am. J. Vet. Res..

[23] Matthews A.G. (1982). Serum protein electrophoresis in horses and ponies. Equine Vet. J..

[24] Arfuso F., Piccione G., Guttadauro A., Monteverde V., Giudice E., Giannetto C. (2023). Serum C-reactive Protein and Protein Electrophoretic Pattern Correlated with Age in Horses. J. Equine Vet. Sci..

[25] Badr H., Young P.E., Dong J., Okorodudu A.O. (2024). Combined bisalbuminemia and Bisalbuminuria: A rare finding on serum and urine electrophoresis. Clin. Chim. Acta.

[26] Kapatia G., Wadhwa M., Malhotra P., Prakash G., Aggarwal R. (2021). Bisalbuminemia: A Pathologist’s Insight of an Uncommon Phenomenon. J. Lab. Physicians.

[27] Kalambokis G., Kitsanou M., Kalogera C., Kolios G., Seferiadis K., Tsianos E. (2002). Inherited bisalbuminemia with benign monoclonal gammopathy detected by capillary but not agarose gel electrophoresis. Clin. Chem..

[28] Mangiagalli G., Meazzi S., Giordano A., Rossi S. (2023). Spurious capillary zone electrophoresis pattern in hypercholesterolemic dogs. J. Vet. Diagn. Investig..

[29] Fayos M., Couto C.G., Iazbik M.C., Wellman M.L. (2005). Serum protein electrophoresis in retired racing Greyhounds. Vet. Clin. Pathol..

[30] Martínez-Subiela S., Tecles F., Montes A., Gutierrez C., Ceron J.J. (2002). Effects of haemolysis, lipaemia, bilirubinaemia and fibrinogen on protein electropherogram of canine samples analysed by capillary zone electrophoresis. Vet. J..

